# The Use of Microelectrodes in Molten Salt Electrochemistry

**DOI:** 10.1021/acs.analchem.6c02514

**Published:** 2026-06-13

**Authors:** Changkyu Kim, Jagadeesh Sure, Adrien Couet

**Affiliations:** Department of Nuclear Engineering and Engineering Physics, 5228 University of Wisconsin-Madison, Madison, Wisconsin 53706, United States

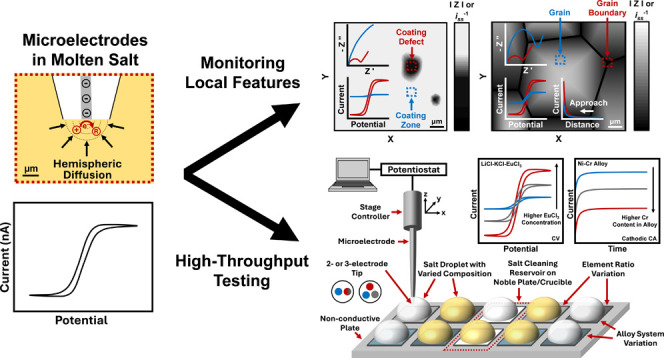

## Introduction

Molten salts have attracted considerable interest as essential media for advanced high-temperature technologies, including molten salt reactors, thermal energy storage, high-temperature electrolysis, and pyrochemical processing.^1–3^ Their ability to remain stable in liquid form at elevated temperatures, combined with favorable thermophysical properties and wide electrochemical windows, makes them highly suitable for applications involving heat transfer, energy storage, and high-temperature electrochemical processing.^4,5^ However, despite these advantages, molten salts present challenges due to their chemically reactive nature at high-temperatures, especially in the presence of oxidizing impurities. Salt chemistry can fluctuate through interactions with impurities over time, or fuel burnup in the case of molten salt reactors, often leading to the dissolution of metal species.^6^ This dynamic environment not only results in complex redox behavior but also promotes corrosion, which is rarely uniform and frequently manifests as localized degradation driven by structural materials’ compositional differences, electrochemical imbalances, and microstructural susceptibilities.^7,8^

Indeed, these corrosion and redox processes in molten salt environments are often localized, initiating at specific microstructural features such as grain boundaries, inclusions, or phase interfaces.^9,10^ Additionally, variations in salt composition near corroding material surfaces can create microenvironments where electrochemical conditions differ significantly from the bulk. As a result, degradation processes such as selective leaching, intergranular attack, and localized deposition often develop in spatially confined regions where localized electrochemical conditions dominate.^11^ Conventional electrochemical techniques, which primarily rely on macroelectrodes, provide bulk-averaged data that can obscure these microscale phenomena.^12^ This limitation makes it difficult to electrochemically probe degradation mechanisms or capture dynamic changes in redox behavior at critical sites, highlighting the need for measurement techniques capable of resolving electrochemical processes at the microscale.

Monitoring these localized electrochemical phenomena in molten salt environments remains a significant challenge. One approach to address this need is the use of microelectrodes, which offer intrinsic advantages for probing site-specific electrochemical phenomena.^13^ Their small dimensions (typically less than 25 μm diameter) offer high spatial resolution, enhanced mass transport, and reduced capacitive and ohmic effects compared to conventional macroelectrodes.^14^ These characteristics allow microelectrodes to detect subtle redox fluctuations and site-specific corrosion behavior that are often undetectable with bulk measurement techniques. As a result, microelectrodes could provide a powerful platform for investigating electrochemical processes in molten salt environments, where localized reactions and dynamic redox shifts are critical yet remain difficult to monitor with conventional methods.

In recent years, microelectrodes have been increasingly explored for electrochemical applications in molten salt environments, with various studies investigating their applicability across different materials, designs, and measurement strategies.^15–18^ Efforts have focused on optimizing electrode fabrication to withstand high-temperatures and aggressive molten salt environments, particularly in relation to electrode sheathing and insulation for localized measurements. Exploratory studies have begun to investigate the applicability of microelectrodes in areas such as scanning probe techniques, confined-volume electrochemistry, and conceptual high-throughput screening strategies, reflecting a growing interest in extending their use beyond conventional bulk analysis.^19–21^ As these efforts continue, a comprehensive and molten salt specific understanding of microelectrode behavior, fabrication challenges, and potential application pathways will be critical to advancing electrochemical monitoring capabilities in complex molten salt systems.

This review summarizes recent progress and ongoing research on microelectrode technologies for high-temperature molten salt environments, highlighting their fundamental merits, material considerations, and emerging strategies for spatially resolved electrochemical monitoring under extreme conditions.

## Microelectrodes as Analytical Tools in Molten Salt Electrochemistry

Traditional electrochemical studies in molten salt systems have largely relied on macroelectrodes, which can be effective for bulk analysis but show inherent limitations in capturing localized or early stage electrochemical phenomena. As illustrated in Table 1, macroelectrodes are constrained by planar diffusion, significant ohmic drop, and slow transient responses, factors that limit both sensitivity and spatial resolution. These issues are particularly pronounced in molten salt environments, where corrosion often initiates at microstructural defects such as grain boundaries or inclusions, regions that macroelectrodes inherently average out, or overlook. In contrast, microelectrodes offer high-resolution, steady-state electrochemical responses with enhanced mass transport and reduced *iR* losses, making them uniquely capable of probing the localized electrochemical dynamics of complex, high-temperature systems.

**Table 1:** General Differences between Macroelectrodes and Microelectrodes

property	macroelectrode	microelectrode
typical dimensions	millimeters to centimeters	micrometers (typically <25 μm)
diffusion profiles	planar; semi-infinite linear diffusion	hemispherical; enhanced radial diffusion
mass transport rate	low	high
current magnitude	μA to mA	pA to nA
steady-state behavior	difficult to achieve by background currents	readily achieves steady-state current
Ohmic drop (*iR*loss)	significant, especially in low-conductivity media	minimal; reduced by small current and localized geometry
response time	slow (ms to hundreds of ms)	fast (ns to μs)
signal-to-background ratio	often low; in dilute or low-conductivity systems	high; due to reduced capacitive and solution resistance effects
spatial resolution	poor; reflects average surface behavior	high; capable of detecting local, site-specific activity

Rather than simply serving as miniaturized alternatives, microelectrodes represent a fundamentally distinct analytical methodology for molten salt electrochemistry. In high-temperature halide melts, key measurement objectives include controlling and quantifying redox speciation, detecting dilute corrosion-driven species against large background currents, and resolving spatially heterogeneous current distributions near material surfaces. For example, during the corrosion of alloys, preferential dissolution of certain phases or grain boundaries may initially generate only trace concentrations of ions or complexes within the near-interface region, while the measured current remains dominated by background contributions. Detecting these weak faradaic signals is mechanistically important because it reveals not only how corrosion reactions initiate locally, but also how the resulting species accumulate, evolve, and eventually perturb the bulk salt chemistry. The following sections therefore frame microelectrode behavior in terms of these molten salt-specific analytical needs, describing how enhanced mass transport, reduced capacitive and ohmic distortions, and microscale geometry enable measurements that are impractical or inaccessible with conventional macroelectrodes.

### Electrochemical Measurement Constraints in Molten Salt Environments

Electrochemical measurements in molten salt environments impose stringent analytical requirements owing to elevated operating temperatures (typically 400 to 800 °C), evolving salt chemistry, and strong spatial gradients in redox conditions near reactive interfaces. Under such conditions, accurate potential control, detection of dilute redox species, and spatial resolution are often required concurrently, particularly when tracking time-dependent changes. Conventional macroelectrodes therefore tend to provide only bulk-averaged information and can suffer from distortions arising from solution resistance, large background currents, and poorly controlled mass-transport regimes.^22^ Microelectrodes, by contrast, function as enabling analytical tools that directly address these constraints.

Although molten salts typically exhibit relatively lower solution resistance than aqueous electrolyte, typically in the range of 1 Ω because of their elevated operating temperatures and large concentrations of charge carriers, ohmic distortions can still become non-negligible in practical experimental geometries.^23^ When large area macroelectrodes are employed, the resulting mA-scale faradaic currents can lead to appreciable potential losses across the electrolyte, distorting cyclic voltammetry (CV) waveform and complicating quantitative extraction of equilibrium potentials or transport parameters.^24^ These effects are exacerbated when reference electrodes cannot be positioned close-enough to the working electrode or when fast potential sweeps or pulsed techniques are used. Accordingly, compensation for ohmic drop is commonly achieved in practice by measuring solution resistance using the high-frequency intercept obtained from electrochemical impedance spectroscopy (EIS).^25^

Elevated temperature further amplifies background contributions at macroelectrodes through increased double-layer charging, leakage currents or overlapping currents from other redox-active species.^12,26^ Because the redox systems of interest, corrosion-generated species or intentionally added redox mediators, are often present at low concentrations, their faradaic responses can be masked by these background currents. As a result, the voltammetric response of macroscopic electrodes may increasingly reflect background contributions rather than the electrochemical processes of interest, thereby reducing sensitivity to early stage corrosion or subtle changes in molten salt chemistry. For example, it has been observed that low-concentration CeCl_3_ in LiCl–KCl–UCl_3_–CeCl_3_ melts could not be reliably quantified by conventional CV, despite the separation in reduction potential, because its signal was greatly obscured and appeared only as a small feature on a large cathodic background dominated by uranium reduction.^27^

Mass-transport regimes at macroelectrodes in molten salt environments can also deviate from the idealized planar diffusion assumed in classical analyses. Strong thermal gradients with convection, as well as increased diffusion coefficients and gas evolution at high-temperatures, disrupt the formation of stable diffusion layers, leading to scan-rate dependent distortions and increased uncertainty in the precise estimation of diffusion coefficients or reaction kinetics from bulk measurements. These complications further constrain the reliability of macroelectrode-based electroanalysis in molten salt environments.

### Analytical Advantages of Microelectrodes in Molten Salt Environments

The diffusion regime at the electrode surface governs the quantitative extraction of transport and kinetic parameters. While macroelectrodes exhibit predominantly planar, transient diffusion,^28^ microelectrodes rapidly establish radial or hemispherical steady-state diffusion regimes^29,30^ due to their small characteristic radius (typically less than 25 μm).^14^ Indeed, this geometric difference enables either a time-invariant, steady-state limiting current when three-dimensional diffusion field is established (such as at an inlaid disk microelectrode), or a quasi-steady-state response that appears nearly constant over practical time windows but can remain weakly time-dependent, such as in recessed or partly shielded microelectrodes where diffusion is not purely radial or hemispherical. As a result, microelectrodes attain steady (or quasi-steady) responses on short time scales, with reduced sensitivity to scan rates and moderate convective disturbances, conditions that may be difficult to maintain at macroelectrodes in high-temperature melts.^31^ The more efficient replenishment of electroactive species from the surrounding electrolyte stabilizes mass transport and improves the reliability of diffusion and kinetic analyses in molten salt environments.

The attainment of steady-state behavior further simplifies quantitative interpretation. Under radial diffusion, the current can be directly related to bulk concentration and diffusion coefficient through established analytical expressions for various microelectrode geometries, as illustrated in Table 2,^14,32,33^ and the symbols are consolidated in the Appendix. In contrast to transient planar diffusion at macroelectrodes, where fitting procedures are often required for CV, steady-state responses reduce ambiguity in parameter extraction. This distinction becomes especially important in high-temperature molten salt environments, where elevated diffusion coefficients and low redox species concentrations often yield weak or rapidly decaying faradaic signals at macroelectrodes. The stable steady-state currents of microelectrodes therefore provide improved signal-to-background characteristics and enhanced detection limits.

**Table 2:** Steady-State or Quasi-Steady-State Equations for Microelectrodes with Different Geometries^14,32,33^

geometry	equation
disk	*i*_ss_ = 4*n*FDCr
hemisphere	*i*_ss_ = 2π*n*FDCr
sphere cap	*i*_ss_ = *kn*FDCr, 4 < *k* < 2πln 2
cylinder	$i_{qss}=\frac{2nFADC}{r\ln\left(4Dt/r^{2}\right)}$
band	$i_{qss}=\frac{2\pi nFADC}{w\ln\left(64Dt/w^{2}\right)}$

A simple back-of-the-envelope estimate helps illustrate these differences. For microelectrodes, the characteristic time required to establish steady-state scales approximately as *t* ∼ *r* ^2^/*D*.^34^ For a representative molten salt diffusion coefficient of *D* ∼ 10^–5^ cm^2^/s, an inlaid disk microelectrode of radius 25 μm can reach steady-state in roughly 10^–1^ to 1 s. By contrast, millimeter-scale macroelectrodes generally do not achieve a true steady-state under quiescent conditions, as their response is governed by planar diffusion, for which the diffusion layer continuously expands, and the current evolves as a transient rather than approaching a constant steady-state value. The corresponding diffusional time scale is instead on the order of 10^2^ to 10^3^ s, far longer than that required for microelectrodes and consistent with the persistence of peak-shaped voltametric responses.

The difference is also evident in the current scale. In the case of an inlaid disk microelectrode, steady-state limiting current is given by *i*_ss_ = 4*n*FDCr, as illustrated in Table 2, whereas the capacitive charging current scales as *i*_c_ = *C*_dl_ *Av*, where *C*_dl_, *A* = π*r* ^2^, and *v* are double layer capacitance, area, and scan rate, respectively. This illustrates that signal-to-background ratio, *i*_ss_/*i*_c_, improves approximately as 1/*r*, because capacitive currents scale with electrode area (∝ *r* ^2^), whereas faradaic currents for general microelectrodes scale approximately with electrode radius (∝ *r*). Therefore, decreasing electrode dimensions produces more significant reduction in background charging relative to faradaic signal.^34,35^ For a redox species at 10^–3^ M with *D* ∼ 10^–5^ cm^2^/s, the microelectrode with 25 μm diameter gives steady-state current of about 5 nA when *n* = 1. Using a representative double-layer capacitance of 20 μF/cm^–2^ in molten salt environments^36^ and a general scan rate of 0.1 V/s, the corresponding charging current is only ∼10 pA, giving a signal-to-background ratio on the order of 10^2^. By comparison, a macroelectrode with an area of 0.5 cm^2^ results in charging current of ∼1 μA under the same condition, which is already larger than the entire steady-state signal of the microelectrode. Although the faradaic current at the microelectrode may be larger in absolute magnitude, the much larger nonfaradaic background makes small concentration changes or weak corrosion-derived signals much more difficult to resolve.

Similarly, ohmic losses (*iR*), which also increase with current, are intrinsically suppressed at microelectrodes due to their low current output.^37^ Ohmic distortions become limiting when large-area electrodes generate currents sufficient to produce potential drops comparable to the intrinsic redox overpotentials being measured, particularly in cells with extended working-reference electrode separation or during fast potential sweeps and pulsed techniques. For example, when *iR* losses approach several tens of millivolts, comparable to peak separations or kinetic overpotentials in high-temperature halide melts, quantitative extraction of thermodynamic parameters becomes ambiguous at macroelectrodes, whereas microelectrodes typically remain within a negligible *iR* regime due to their reduced current scale. These scaling relationships clarify when microelectrodes provide analytical advantages in molten salt environments, as schematically illustrated in Figure 1.

**Figure 1:**
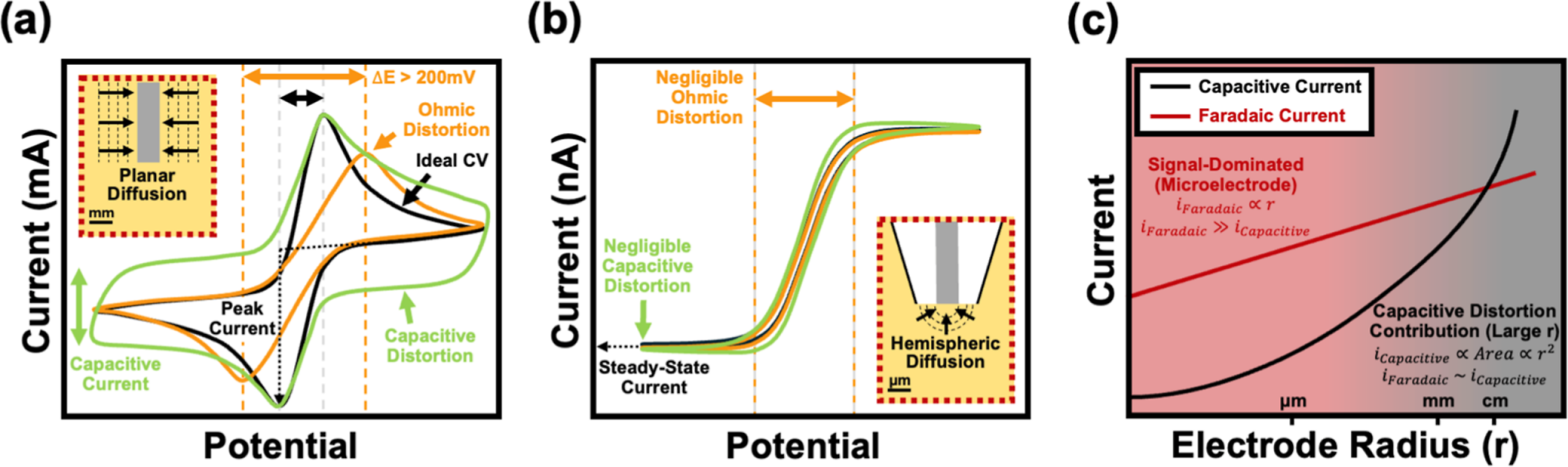
Schematic comparison of cyclic voltammograms at (a) macroelectrodes and (b) microelectrodes, highlighting ohmic and capacitive distortions, and (c) the radius-dependent scaling of faradaic and capacitive current contributions.

Beyond bulk parameter estimation, the confined diffusion fields associated with microelectrodes also enable investigation of spatially localized electrochemical environments.^38,39^ The small sampling volume adjacent to the microelectrode surface makes it possible to probe redox gradients near reactive interfaces, such as corroding grains or alloy boundaries, without averaging over the entire melt. A useful estimate of the spatial sampling scale for microelectrodes has been suggested from an effective-volume model in molten salt environments.^40^ In this model, the sampling volume is assumed to be spherical, and measured steady-state current of the inlaid disk microelectrode is related to the average concentration within that volume driven by the actively corroding surface. Using such relationships, the sampling radius was estimated to be approximately 3.2*r* to 3.7*r*, where *r* is the microelectrode radius, depending on probe-substrate distance.

On this basis, a 25 μm diameter microelectrode (*r* = 12.5 μm) corresponds to a sampling radius of roughly 40 to 46 μm. Such a scale maybe therefore well matched to probe spatially heterogeneous electrochemical environments, such as locally corroding regions, grain-scale compositional variations, or concentrated redox gradients near reactive interfaces. However, grain boundary electrochemical activity could only be detected if grain sizes are larger than 100 μm. Reducing the microelectrode radius further should proportionally contract the sampling volume, thereby enabling more spatially selective measurements and, in principle, access to correspondingly smaller features. In this way, microelectrodes offer not merely an alternative diffusion geometry, but a distinct analytical modality tailored to the spatial and temporal complexities of molten salt systems.

Overall, the analytical value of microelectrodes in molten salt environments lies not only in their current scaling, but also in their ability to probe distinct local electrochemical regimes. By allowing radial diffusion, suppressing ohmic and capacitive distortions, and minimizing the sampling volume, microelectrodes enable electrochemical measurements that would otherwise remain obscured or strongly averaged at conventional macroelectrode dimensions. In this sense, microelectrodes function not merely as miniaturized macroelectrodes, but as analytical tools readily probing subtle and localized phenomena evolving in molten salt environments.

## Practical Implementation of Microelectrodes in High-Temperature Molten Salt Environments: Challenges and Mitigation Strategies

The analytical capabilities of microelectrodes establish a compelling framework for molten salt analysis. However, translating these capabilities into high-temperature experiments requires careful consideration of both intrinsic electroanalytical constraints and practical implementation challenges. Molten salts, typically operating above 500 °C, constitute chemically aggressive ionic media, especially when trace impurities, such as oxygen, moisture, or redox-active species, are present which can significantly increase corrosivity. In such systems, both metallic electrodes and insulating components are exposed to sustained thermal and chemical stressors. Under such conditions, electrode materials may undergo oxidation, dissolution, or embrittlement, particularly in the presence of reactive redox species, while insulating materials used to define microscale geometries, typically glass, can experience thermal expansion mismatch, cracking, or chemical degradation during prolonged exposure. As a result, some limitations arise from high-temperature ionic media in the presence of impurities, whereas others stem from materials selection, fabrication approaches, and mechanical integration. Distinguishing between these categories is essential for clarifying which constraints reflect inherent features of molten salt electrochemistry and which can be mitigated through improved design and engineering strategies.

### Microwire Compatibility

Microwires constitute the conductive core of microelectrodes and therefore directly influence the accessible electrochemical window and measurement reliability in molten salt environments. Material selection is particularly critical under high-temperature conditions, where chemical reactivity, alloy formation, and electrochemical dissolution can alter surface behavior during operation. Since microelectrode responses are highly sensitive to surface state, even minor alloy-induced compositional changes can lead to irreversible peak shifts, hysteresis, and altered interfacial kinetics. As a result, microwire elements that have been commonly employed in aqueous electrochemistry, such as platinum (Pt) or gold (Au), may impose electroanalytical limitations when reactive metallic species are present, compromising their electrochemical reliability.

In molten salt electrochemistry, dissolved metallic species such as Ni, Cr, Fe, Sn, Al, Pb, or Na may be present in the melt, and Pt- or Au-based microwires may thermodynamically undergo surface alloying with these species.^41–48^ Specifically, Pt has been shown to alloy with dissolved Fe, Al, and Na in molten salt environments,^49,50^ whereas Au has been suggested to alloy similarly with Al and Sn,^51^ producing distinctive peaks during CV scans.

Refractory metals such as tungsten provide an alternative with distinct advantages under molten salt environments. Tungsten exhibits not only high temperature compatibility as it has the highest melting point among metals, but also in many molten salt systems, a comparatively wider electrochemical stability window compared to Pt or Au.^40^ In a typical molten chloride system, such as eutectic LiCl–KCl, the practically accessible potential window is roughly ∼3.5 V for W, whereas it is narrower for Pt ∼ 1.5 V and Au ∼ 2.0 V, likely due to the electrochemical dissolution or alloying tendencies with lithium.^40^ Unless oxygen species are present at sufficiently high levels to narrow the potential window through tungsten oxide formation, as in melts with intentional oxygen addition, tungsten remains highly stable over a broad potential range in typical well-controlled environments including glovebox setups, often up to chlorine gas evolution.

Thus, microwire compatibility is defined by the potential range over which it remains chemically and electrochemically stable in the molten salt without surface modification or degradation. Appropriate microwire selection is therefore essential to maintain microelectrode integrity and ensure that the measured response remains representative of the redox chemistry of interest.

### Insulation Stability and Electrochemical Constraints

The insulating sheath or coating layer of a microelectrode defines the exposed active area and provides electrical insulation from the surrounding melt. In aqueous environments, glass-based insulations, such as borosilicate (also known as Pyrex) and quartz (also known as fused silica), are widely used for insulating microwires due to their chemical stability, thermal resistance, and ability to be shaped into customized geometries.^52–54^ However, in high-temperature molten salt environments, their stability varies depending on the specific applications, with borosilicate and quartz offering distinct advantages and challenges.

Borosilicate glass is commonly chosen for microelectrode fabrication owing to its high thermal resistance and softening temperature (∼800 °C), which allows reliable performance in most high-temperature environments. It is also chemically compatible with commonly used molten chloride salts, such as LiCl–KCl,^55,56^ and relatively easy to process, making it a practical choice to manufacture precise electrode geometries. Despite these advantages, borosilicate has notable drawbacks. Prolonged exposure to molten salts can enhance alkali-ion mobility in borosilicate-based insulation, which can compromise chemical stability and may promote the introduction of sodium- or potassium-containing impurities into the salt, especially at elevated temperatures higher than ∼485 °C.^21,57^ Additionally, the mismatch in thermal expansion coefficients between borosilicate and the underlying microwire can induce stresses during thermal cycling, leading to interface cracking or delamination,^58,59^ reducing the overall durability of the microelectrode. Its inherent brittleness further increases the risk of mechanical failure, particularly under electrode motion.

Another primary concern is insulation-driven currents. Glass-based insulations, particularly borosilicate, exhibit increased capacitive-like behavior, partly associated with the mobility or dissolution of alkali species within the glass matrix. As temperature increases, an intrinsic glass electrode current (IGEC) becomes measurable even in the absence of electroactive species. In microelectrode configurations, where steady-state faradaic currents scale with electrode radius and are therefore inherently small, such nonfaradaic contributions can become non-negligible. Consequently, the presence of IGEC may distort electrochemical measurements, such as CV.

Specifically, as shown in Figure 2a–c, CV curves obtained using borosilicate-based microelectrode in LiCl–KCl with the absence of electroactive species exhibit temperature-dependent currents that increase with temperature.^21^ At elevated temperatures, the blank response becomes non-negligible compared to the magnitude of the faradaic signal observed for the Eu­(III)/Eu­(II) redox couple. This contribution introduces ambiguity in redox identification, distorts apparent curve shapes, and may lead to overestimation of diffusion coefficients, or misinterpretation of redox reversibility if not properly accounted for. Furthermore, the effect may become especially critical when investigating dilute corrosion-generated species or redox mediators with extremely low concentrations, where the extent of faradaic signal is expected to be small. Such high-temperature-driven current contributions therefore define a fundamental analytical boundary for borosilicate-coated microelectrodes in molten salt systems that needs to be considered in experimental design and data interpretation.

**Figure 2:**
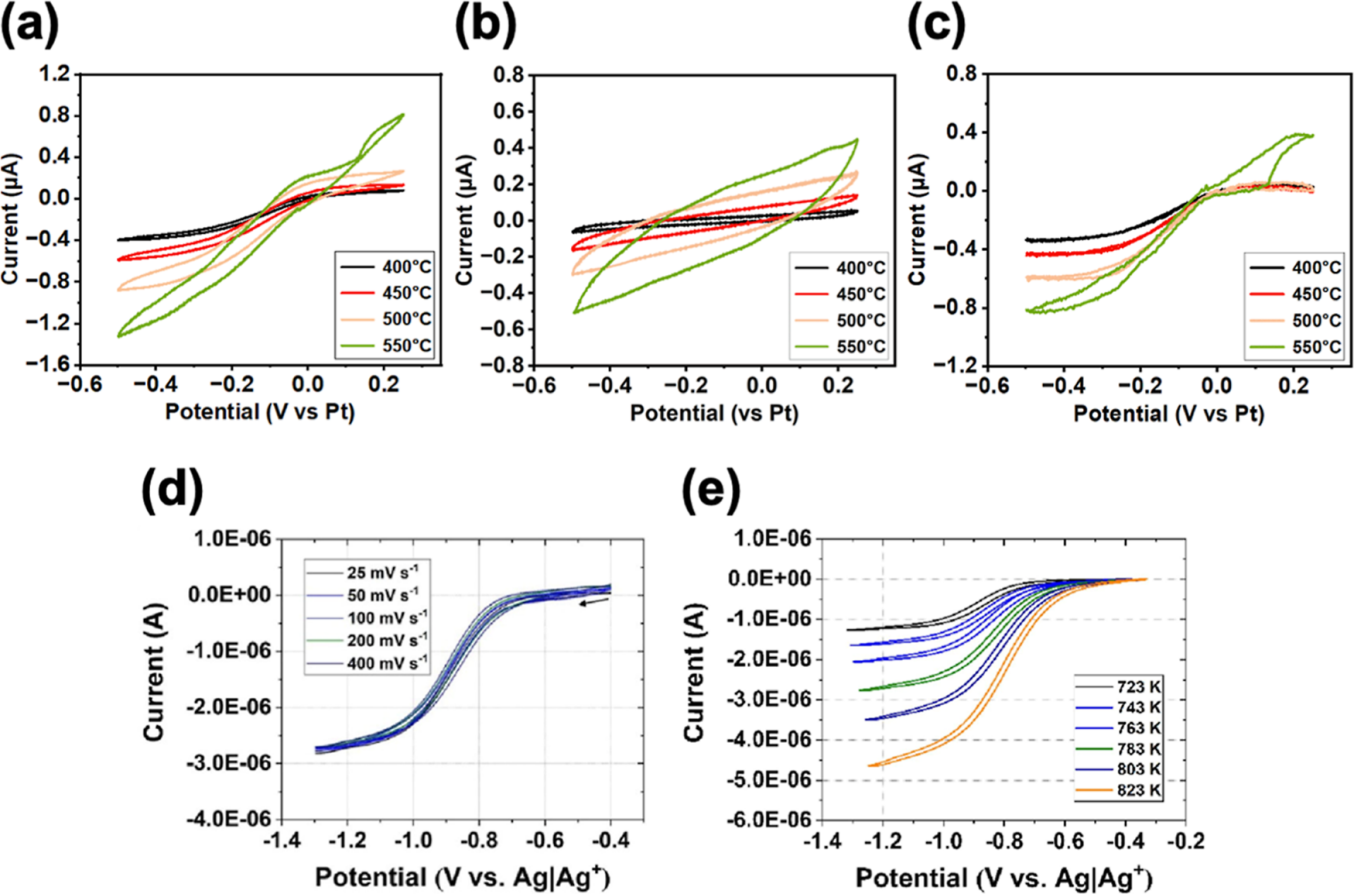
(a–c) CV curves in LiCl–KCl eutectic obtained by borosilicate-insulated 25 μm diameter W microelectrode (a) with and (b) without 2 wt % EuCl_3_ using varied temperatures at 2 mV/s and (c) CV curves after IGEC subtraction. Reproduced with permission from ref 21. Copyright 2025 American Chemical Society. (d,e) CV curves in LiCl–KCl eutectic obtained by quartz-insulated 50 μm diameter W microelectrode (a) with 2.1 wt % SmCl_3_ using varied scan rates at 773 K and (b) with 1.8 wt % SmCl_3_ using varied temperatures at 25 mV/s scan rate. Reproduced with permission from ref 16. Copyright 2023 Elsevier.

Quartz provides relatively improved performance in chemically aggressive high-temperature molten salt environments compared to borosilicate glass. With a thermal stability exceeding 1000 °C, quartz is highly resistant to structural degradation in high-temperature environments. Unlike borosilicate glass, which contains mobile alkali ions, such as Na^+^ and K^+^, that may dissolve into molten salts and increase conductivity of the insulation sheath at high-temperatures, quartz is composed of pure silica which is free of such species. This results in relatively lower conductivity and greater chemical stability in molten chloride salt environments, making quartz a more inert candidate. Figures 2d,e demonstrate that quartz-coated microelectrodes can maintain electrochemical stability and signal integrity across a wide range of temperatures and scan rates in LiCl–KCl containing SmCl_3_ with relatively smaller IGEC than borosilicate-coated microelectrodes.^16^

Despite its chemical stability advantages, the low coefficient of thermal expansion of quartz still introduces interfacial stresses when paired with microwire materials, such as tungsten. This mismatch can lead to localized mechanical stress accumulation at the interface during thermal cycling, resulting in microcracks or delamination, especially under rapid temperature changes during either heating/cooling of the furnace or handling of microelectrodes. Moreover, the fabrication of quartz coatings is relatively more challenging compared to borosilicate because of its higher softening and melting temperatures, often necessitating vacuum conditions to prevent microwire oxidation during application. These factors increase production and application complexity and cost, making quartz less practical. Additionally, despite its high thermal and chemical stability, quartz is more brittle than borosilicate,^60^ requiring even more careful handling to avoid mechanical damage during fabrication or use.

Meanwhile, the glass-based insulations cause another electroanalytical constraint arising from the electrochemical stability window of the insulation itself. Recent electrochemical studies have revealed nuanced differences in the reduction behaviors of borosilicate and quartz glasses under CaCl_2_ environments,^61,62^ as illustrated in Figure 3. In molten CaCl_2_ at 1123 K, it has been demonstrated that both borosilicate and quartz can undergo electrochemical reduction by applied electrochemical potential, yet borosilicate showed relatively less severe surface degradation, but deeper degradation compared to quartz. This trend is attributed to the preferential and deeper bulk reduction of B_2_O_3_, resulting in relatively limited surface-level changes, as confirmed by optical microscopy and XPS analyses. In contrast, quartz underwent more surface reductions, leading to visibly greater surface degradation despite its lower reduction currents.

**Figure 3:**
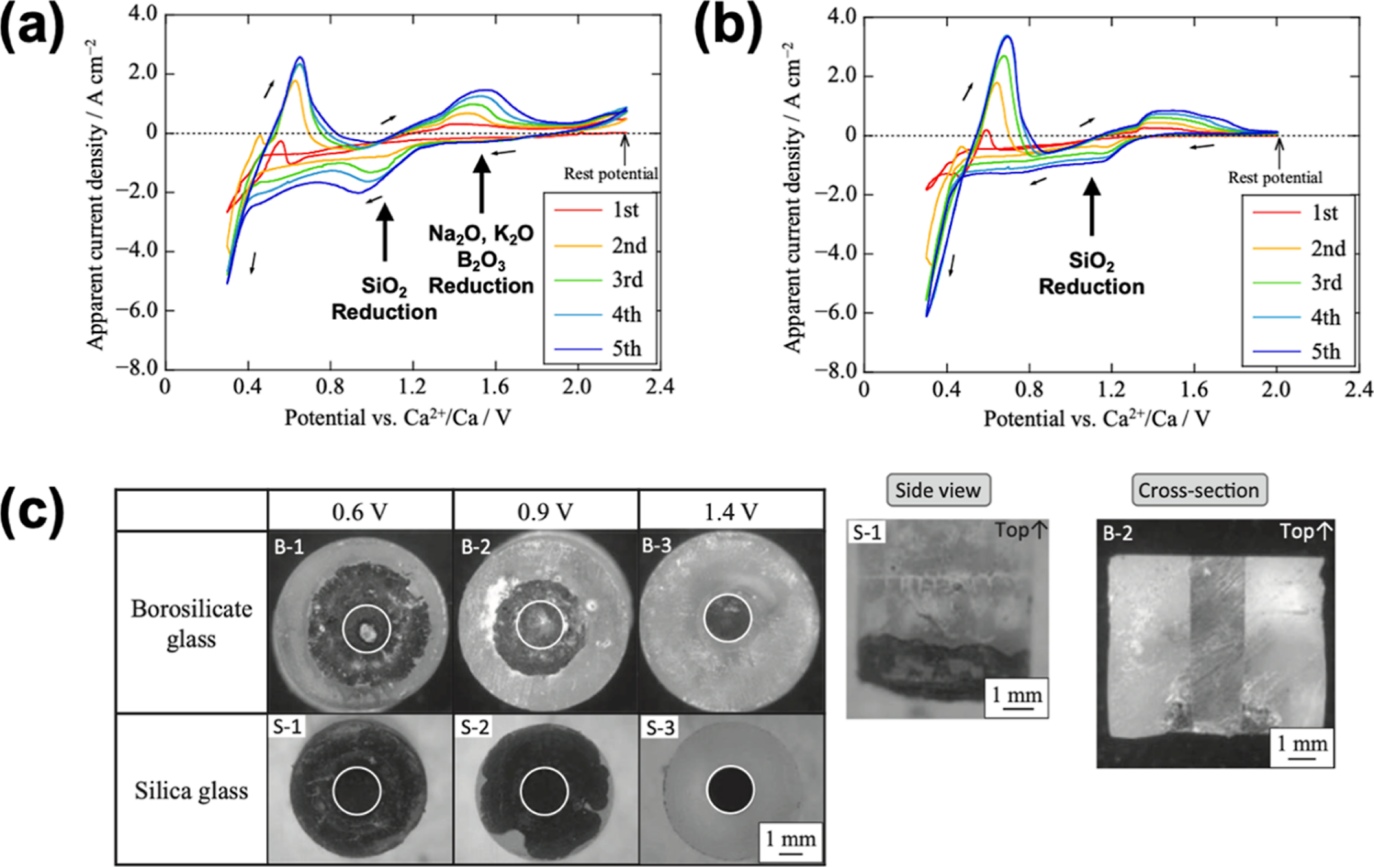
Cyclic voltammograms for sealed (a) borosilicate and (b) quartz electrodes in molten CaCl_2_ at 1123 K with 100 mV/s scan rate. (c) Microscopy images of the sealed glass electrodes following potentiostatic electrolysis at varied applied potentials vs Ca­(II)/Ca for 30 min in molten CaCl_2_ at 1123 K. White circle shows the boundary between the tungsten rod and the glass. Reproduced with permission from ref 61. Copyright 2016 The Electrochemical Society.

These findings suggest that the use of glass as an insulation material generates cathodic stability limits due to the electrochemical reduction of glass-composing oxides, such as SiO_2_ or B_2_O_3_, in high-temperature molten salt environments. When operating potentials approach these limits, partial reduction of the insulating material can occur, narrowing the useable electrochemical window and introducing additional currents nonrelevant to targeted redox couples. As a result, certain lanthanide redox couples (such as La, Ce, Pr, Nd, or Gd) may fall beyond the stability boundary of glass-coated microelectrodes, constraining accessible electrochemical space independent of microwire selection. These relative stability limits with representative redox couples are schematically illustrated in Figure 4. This calls for the development of alternative coating materials with broader electrochemical stability to enable more comprehensive studies of lanthanide and actinide redox reactions.

**Figure 4:**
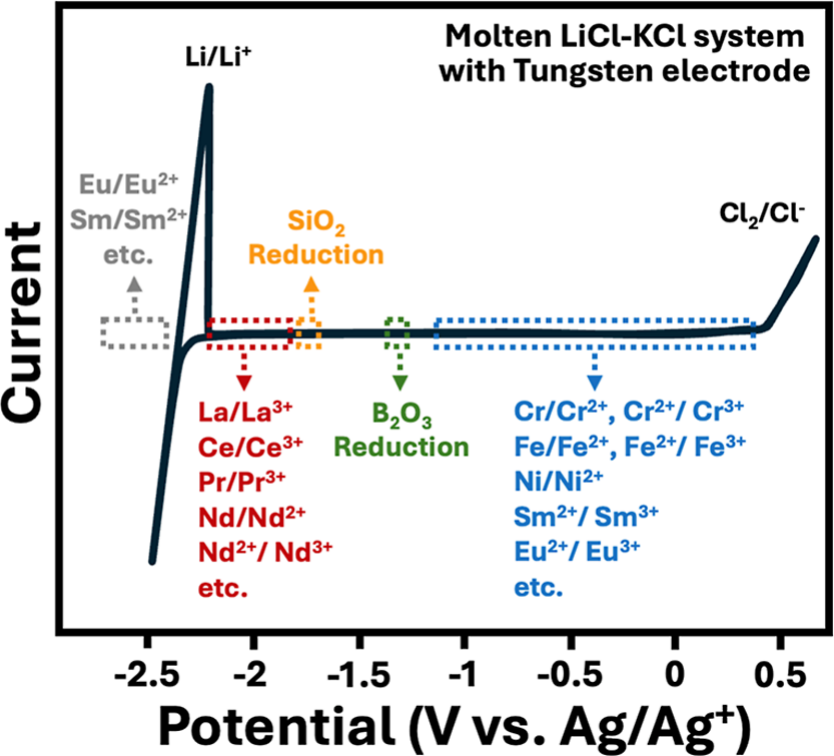
Schematic representation of the electrochemical stability limits of glass-coated tungsten microelectrodes in molten LiCl–KCl. The overlapping potential ranges highlight the representative elements of interest in high-temperature molten salt corrosion studies, highlighting the limited accessibility of certain redox couples due to early onset of glass reduction, thereby restricting the useable potential window for electrochemical measurements.

To overcome these limitations and constraints in electroanalytical analyses, ceramic insulations have emerged as a promising alternative to traditional glass-based insulations for microelectrodes. Their superior chemical inertness, thermal stability, and resistance to degradation under aggressive electrochemical conditions make them suited for applications involving the electrochemical study of targeted redox elements. Recent advances in materials science and manufacturing techniques have enabled a growing interest in the use of ceramics for microelectrode applications in molten salt environments. Importantly, ceramics such as boron nitride (BN) have demonstrated chemical compatibility even with molten fluoride salts, including FLiNaK and FLiBe systems.^63,64^ Consistent with this, BN has also remained chemically inert as an insulating sheath during EIS measurements in FLiNaK environment, although the electrode dimensions were larger than those of typical microelectrodes.^65^ Their demonstrated stability in such environments suggests that ceramic-based microelectrode may therefore open the possibility of extending electroanalytical techniques to fluoride-based molten salts.

Recent developments in ceramic-coated microelectrodes have leveraged established thin-film fabrication techniques to achieve robust operation in molten salt environments. In one reported study shown in Figure 5,^15^ a thin layer of platinum or tungsten was deposited onto a substrate, then encapsulated by insulating films of silicon dioxide (SiO_2_) and silicon nitride (Si_3_N_4_), leaving only a micrometer-scale disk area exposed via photolithographic patterning. This microelectrode design was evaluated in molten LiCl–KCl eutectic salt at temperatures up to 773 K, using CV and chronoamperometry to probe the redox behavior of species such as Zn­(II) and Eu­(III). Under these conditions, the ceramic-encapsulated electrodes were reported to maintain steady-state current responses and exhibit minimal signs of physical degradation during measurement periods exceeding 30 min. These results suggest improved temporal and geometric stability relative to conventional glass-pulled microelectrodes, which showed progressive damage and increased current artifacts under the same conditions.

**Figure 5:**
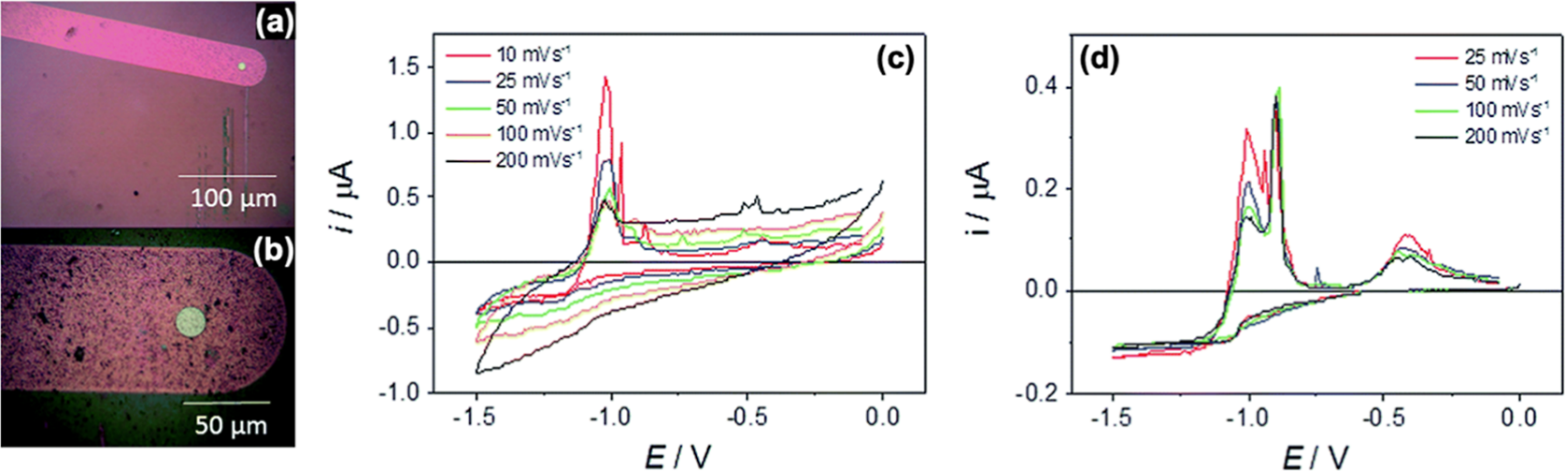
Images of molten salt compatible microelectrodes (a,b) and CV curves in LiCl–KCl eutectic with 5 mM ZnCl_2_ at 773 K obtained with glass pulled tungsten microelectrode (c) and platinum molten salt compatible microelectrode (d). Reproduced with permission from ref 15. Copyright 2016 The Royal Society of Chemistry.

Taken together, insulating materials define a practical balance among fabrication ease, thermal and chemical stability, electrochemical window, and background current in microelectrodes for molten salt environments. These trade-offs ultimately govern measurement fidelity, operational durability, and the range of salt chemistries and redox systems that can be reliably probed. A direct comparison of representative insulating materials is summarized in Table 3.

**Table 3:** Comparative Performance Characteristics of Insulation Materials for Microelectrodes in High-Temperature Molten Salt Environments

property	borosilicate	quartz	ceramic
thermal stability	annealing point ∼565 °C	annealing point ∼1100 °C	annealing point often exceeds 1500 °C
	service range <500 °C	service range <1000 °C	service range >1000 °C
chemical compatibility	generally good	generally better	generally excellent
			resistant to fluoride salt
electrochemical window	limited by both SiO_2_ and B_2_O_3_ reduction	wider than borosilicate, limited by SiO_2_ reduction	generally wider
			
background current (IGEC)	significant at >500 °C	relatively lower than borosilicate	minimal
fabrication complexity	moderate	high	very high
typical failure modes	thermal shock and cracking	thermal shock and cracking	thermal shock and cracking
	leakage of alkali ions	more brittle than borosilicate	

## Application of Microelectrodes to Emerging Electrochemical Platforms and High-throughput Experiments in Molten Salt Systems

The application of microelectrodes in molten salt environment remains relatively recent, but it is rapidly expanding in both scope and sophistication. Initially adopted for fundamental studies of redox couples and diffusion behavior under high-temperature molten salt conditions, microelectrodes are now being explored for more advanced configurations that enable spatial resolution and localized measurements. While localized electrochemical techniques, such as scanning electrochemical microscopy (SECM) and localized electrochemical impedance spectroscopy (LEIS), have been well established in aqueous or low-temperature systems,^66–69^ their application to high-temperature molten salt environments remains limited and largely conceptual. Nonetheless, these techniques serve as valuable references for researchers to explore how spatially resolved electrochemical probing can be adapted for high-temperature environments.

While the implementation of localized electrochemical techniques in molten salt environments remains technically challenging, one notable advantage of many halide-based molten salt systems is their relatively lower volatility at elevated temperatures. This property minimizes solvent loss and allows for more stable confined geometries compared to aqueous systems, where evaporation often limits spatially resolved or long-duration electrochemical experiments. Specifically, compared to the vapor pressure of water at 25 °C, the vapor pressure of molten salts are known to be predominantly lower by about an order of magnitude at their typical operating temperature (500 to 800 °C).^70–72^ In this context, high-temperature platforms utilizing a single molten salt droplet coupled with a microelectrode may offer a practical and experimentally feasible route toward high-throughput electrochemical screening.

### Localized Electrochemical Techniques: Adapting SECM and LEIS to Molten Salt Environments

Localized electrochemical techniques, such as SECM and LEIS, have proven highly effective in aqueous systems for probing redox reactions, corrosion activity, and interfacial charge transfer with microscale resolution.^73^ These techniques rely on the precise control of microelectrode position relative to a reactive surface using an electrode stage controller, enabling the spatial mapping of electrochemical phenomena that are otherwise averaged out in conventional bulk measurements. Translating these capabilities to molten salt environments presents substantial challenges, including the need for high-temperature stability, chemically robust electrode sheathing materials, and reliable positional control.

Despite these obstacles, recent work has demonstrated that SECM-inspired measurements can be realized in molten salt systems.^21^ For example, microelectrode approach curve experiments have successfully distinguished between insulating substrates and actively corroding metal surfaces, with current response patterns consistent with classical negative and positive feedback modes. Particularly, a pronounced increase in current response was observed near corroding Ni–20Cr substrates in LiCl–KCl containing EuCl_3_. This behavior reflects the positive feedback associated with the localized reduction, at the microelectrode, of species released during the Ni–20Cr corrosion process. Moreover, time-dependent approach curves further revealed gradual shifts in the local open-circuit potential, offering spatially and temporally resolved insight into redox activity above the degrading substrate. These findings highlight the potential for developing electrochemical techniques in molten salt environments, enabling activity mapping at the grain or even the grain boundary scale, where variations in crystallographic orientation may govern localized corrosion susceptibility and interfacial redox behavior.

By contrast, to the best of current knowledge, LEIS has not yet been experimentally demonstrated in high-temperature molten salt environments, though its advantages remain compelling. Hence, the discussion below is therefore conceptual and intended to outline its potential applicability rather than describe established implementation. In aqueous systems, LEIS enables localized impedance acquisition directly at the sample surface, providing quantitative insight into coating degradation, interfacial impedance, or localized corrosion resistance.^74,75^ Unlike SECM, which applies potential to the scanning probe and typically yields qualitative information, LEIS applies the potential to the substrate and collects impedance responses via a bielectrode probe. This allows LEIS to yield more direct and quantitative analysis at specific surface regions, where conventional EIS would average out local variation. While its spatial resolution is relatively lower than SECM due to its intrinsic geometry that requires physical separation between the two electrodes of the probe,^76^ LEIS still offers the unique advantage of providing direct quantitative impedance information at localized areas of interest.

If successfully adapted to molten salt environments, this approach could provide quantitative local impedance data at selected surface regions, which would be particularly valuable for detecting localized coating degradation, interfacial failure, or defect-driven corrosion processes in high-temperature systems, where measurable changes in charge-transfer resistance or double-layer capacitance can indicate degraded regions. Given the importance of protective coatings for extending the lifetime of structural alloys in molten salt environments,^63,77^ LEIS complements high-resolution techniques by offering localized impedance data tied to varied surface failure modes, enabling quantitative assessment of interfacial degradation.

A schematic summary of SECM and LEIS setups and representative application scenarios is illustrated in Figure 6. The top panels show the general instrumental configurations of each technique using microelectrodes, while the bottom panels provide representative examples across a shared spectrum of applications. These include localized analysis of interfacial impedance, coating degradation, and site-specific electrochemical activity, where either technique may be selectively employed depending on the desired resolution or quantification need.

**Figure 6:**
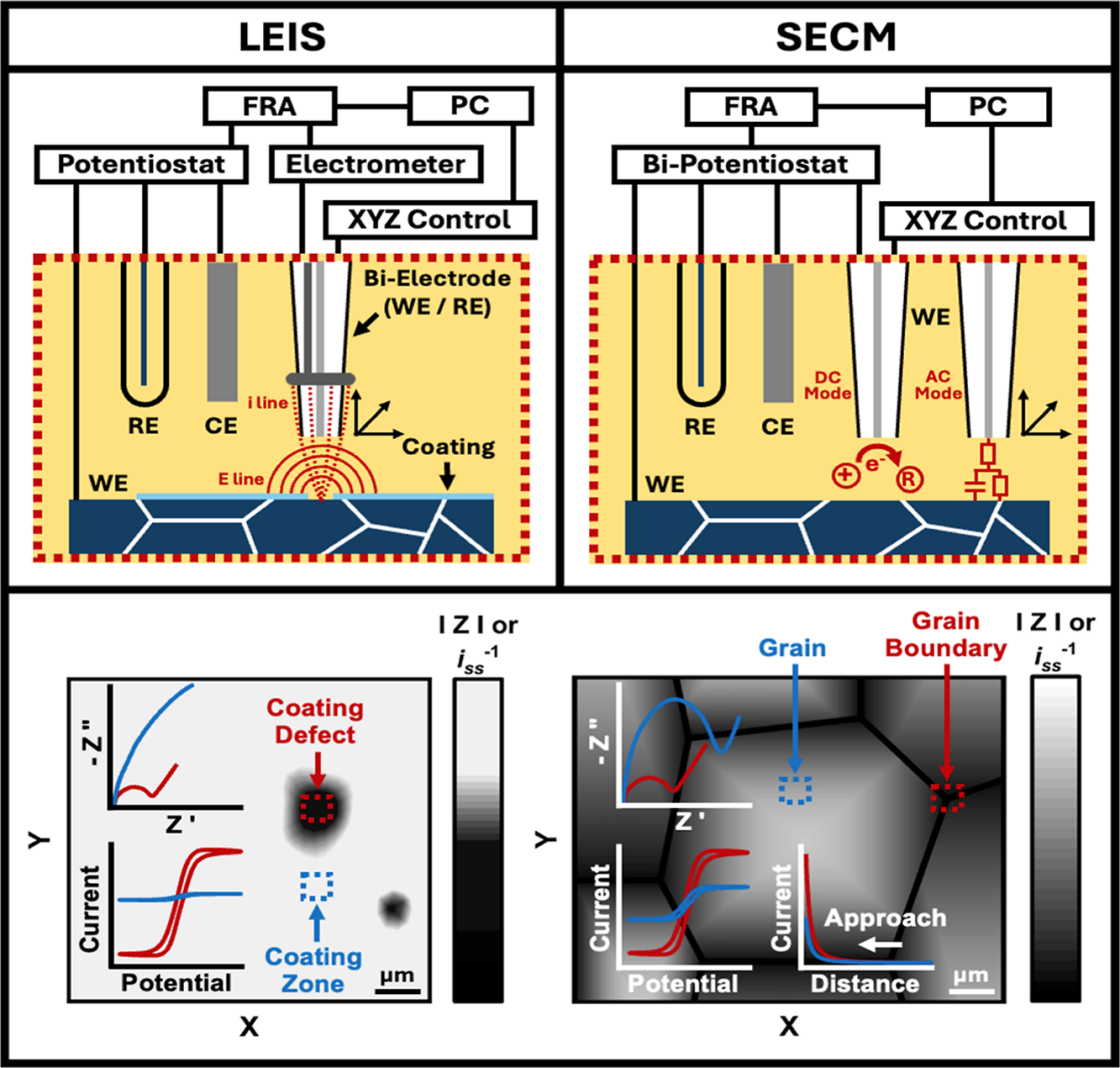
Schematic comparison of LEIS (left) and SECM (right) configurations and representative application scenarios. Top panels depict the respective instrumental setups for each technique using microelectrodes, highlighting probe configuration and signal acquisition modes. Bottom panels present example measurement results of local impedance or current responses for detecting coating defects or grain/grain boundary activities.

### Droplet-Based Electrochemistry and Emerging High-throughput Approaches in Molten Salt Environments

Droplet-based electrochemical systems have been widely utilized in aqueous environments, primarily in the form of microdroplets designed to probe localized electrochemical reactions while minimizing sample volume.^78,79^ These approaches have enabled fine spatial control and have been applied to study redox behavior, surface activity, and interfacial kinetics. However, the high-volatility of aqueous electrolytes imposes severe limitations on droplet stability even under ambient conditions. To overcome such limitations, oil-immersion strategies have been developed to suppress evaporation and extend experimental durations, and have been successfully implemented into the microdroplet electrochemical systems.^80^

In contrast, molten salts possess inherently lower vapor pressure together with relatively better thermal stability, making them naturally suited for confined-volume electrochemistry at high-temperatures.^70–72^ This opens the possibility of using droplet-based configurations without the need for external coverage that prevents their evaporation. When combined with microelectrodes, molten salt droplets could, in principle, support localized redox measurements or compositional screening in a miniaturized format. While the idea of using microdroplet arrays in combination with localized electrochemical techniques is conceptually attractive, such configurations remain challenging in molten salt systems due to difficulties in thermal management, microdroplet preparation and positioning, and need for well-characterized surfaces to allow meaningful comparisons. Although confined-volume setups using microcapillaries may offer one potential solution, their implementation in extreme conditions is constrained by high-temperature compatibility issues, such as sealing gasket materials or pressure control, together with preventing effective use of the key advantages of microelectrodes. Instead, the use of microelectrodes in bulk-droplet systems offers a more practical and scalable approach for high-throughput electrochemical screening, enabling rapid sorting and evaluation of alloy or salt compositions with improved experimental control and reproducibility.

A recent study demonstrated the use of molten salt droplets placed on metallic substrates, wherein a miniaturized electrochemical probe was employed to monitor time-dependent corrosion behavior under high-temperature conditions.^19^ This droplet-based approach highlights the feasibility of performing sequential electrochemical measurements across multiple samples, illustrating how confined molten salt volumes can support high-throughput experiments in molten salt environments.^20,81^ Beyond this immediate implementation, this configuration also offers a modular framework for generating electrochemical data sets across varied alloy compositions together with automated CV analysis, wherein electrochemical signals can be systematically collected and analyzed to assess corrosion related properties.

While the study successfully demonstrated the feasibility of droplet-based electrochemical measurement in a molten salt environment,^19^ it employed a single-droplet configuration with miniaturized macroelectrodes, which may limit quantitative reproducibility due to variations in electrode exposure and geometry, particularly when extended to the high-throughput in situ characterization across multiple samples. A promising advancement of such configuration involves the use of microelectrodes, which inherently feature geometrically defined and fixed surface areas, minimizing uncertainty in signal interpretation. In addition to offering reduced capacitive background, stable steady-state response, and minimal ohmic drop, microelectrodes are more readily integrated with confined molten salt volumes. These attributes would enable efficient, reproducible high-throughput electrochemical screening across diverse salt compositions and alloy systems tailored to extreme environments.

Such concept is illustrated in Figure 7, which shows a representative microelectrode-based setup for high-throughput electrochemical screening using molten salt droplets across varied alloy and salt compositions. This highlights the potential of microelectrodes to transform molten salt electrochemistry into a more modular, scalable, and data-rich platform. Nevertheless, several practical constraints must be considered to ensure data reproducibility and quantitative reliability, owing to the much smaller electrolyte volume, which renders the system particularly sensitive to environmental perturbations. Key practical challenges include precise thermal control of molten salt droplets, maintaining droplet size reproducibility across sequential experiments, and preventing cross-contamination between droplets of different compositions.

**Figure 7:**
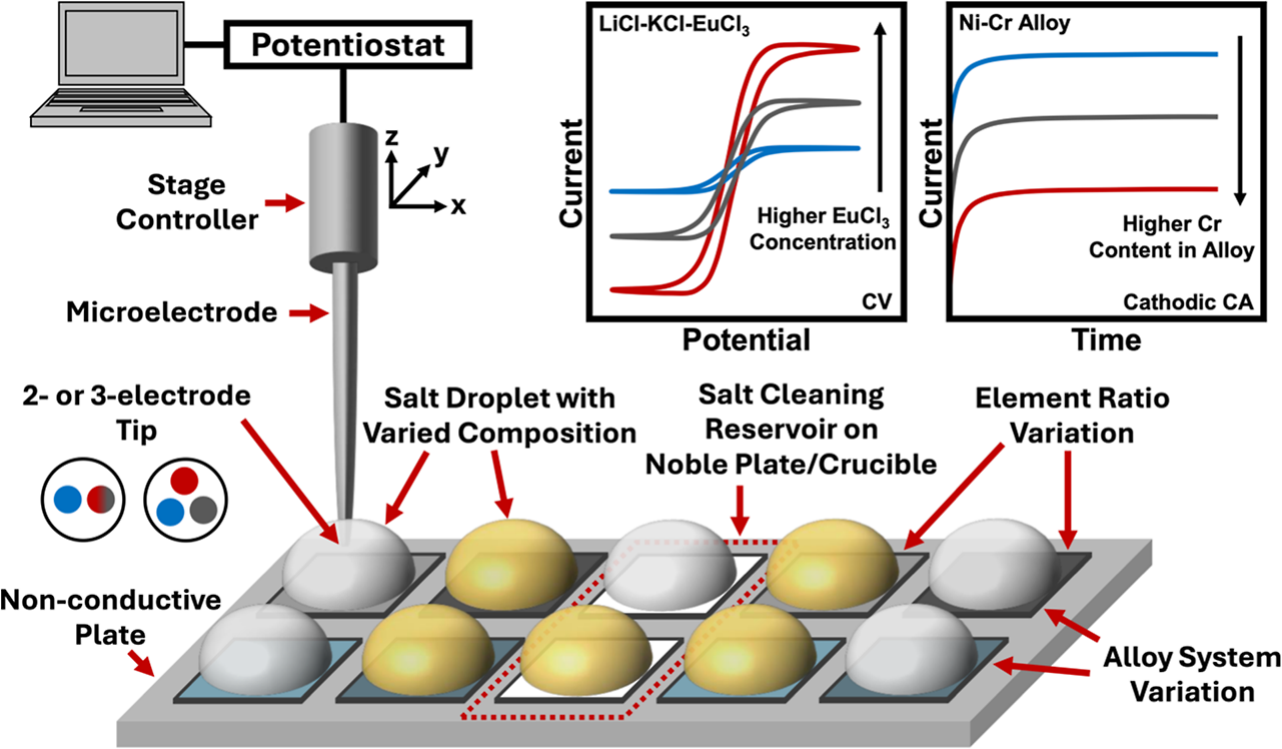
Schematic of high-throughput electrochemical screening platform using microelectrodes and molten salt droplets. Individual droplets placed on an insulating plate enable compositional variation across salt and alloy systems. A stage-controlled microelectrode performs electrochemical measurements in a 2- or 3-electrode configuration. Representative electrochemical signals illustrate trends across different parameter variations.

Precise thermal control can be challenging because high-throughput platforms using molten salt droplets would require repeated microelectrode access and motion, which complicates the use of fully sealed, isothermal cells. As a result, heat losses and local temperature gradients can vary between measurements. A practical mitigation may be employing a temperature-controlled stage that establishes a reproducible thermal boundary condition at the droplet–substrate interface while minimizing convective heat exchange through shielding or controlled atmospheres. Where feasible, brief dwell periods after the microelectrode repositioning may also help re-establish thermal steady-state prior to data acquisition. Droplet size reproducibility can be improved by prepelletizing salts or high-temperature dispensing systems, such as pipettes, that directly introduces molten salt of defined volume on the sample of interest. Such approaches can help stabilizing the effective diffusion geometry across a screening array. Finally, cross-contamination during electrode movements can be minimized by incorporating a cleaning or conditioning reservoir between measurements and applying standardized rinsing and dwell protocols before moving to the next composition. These considerations highlight that droplet-based platforms require careful experimental design to ensure reliable comparison across high-throughput measurements.

## Conclusions

The development of microelectrode technologies offers a practical and powerful approach for improving electrochemical measurements in high-temperature molten salt environments. Microelectrodes address key limitations of conventional macroelectrodes by enhancing mass transport, minimizing capacitive and ohmic interferences, and enabling localized measurements with high spatial resolution, which are critical for investigating redox reactions, ion transport, and microstructurally dependent corrosion behavior under extreme conditions.

While microelectrodes offer clear advantages, considerations regarding material compatibility in high-temperature molten salt environments remain important. Both traditional glass-based coatings and emerging ceramic materials present distinct trade-offs in terms of electrochemical stability and manufacturing complexity. Recent advances in ceramic coatings, although still limited, have demonstrated promising potential to improve the performance of microelectrodes in molten salt environments. These developments, alongside continued refinement of glass-based coating systems, are expected to expand the operational scope of microelectrodes, particularly in applications demanding extended durability and broader electrochemical potential windows under extreme conditions.

In addition to material advancements, the use of microelectrodes in molten salt environments is being extended beyond fundamental redox studies to encompass spatially resolved and confined-volume techniques. Experimental demonstrations, such as high-temperature SECM, have validated the feasibility of spatially resolved measurements under extreme conditions, while conceptual efforts continue for techniques such as LEIS. Moreover, droplet-based approaches have shown potential for high-throughput electrochemical screening, leveraging the low volatility of molten salts to enable stable and confined-volume platforms. These advances further highlight the unique role of microelectrodes in enabling localized, spatially resolved, and quantitative electrochemical analysis in molten salt environments. Although these developments illustrate the growing versatility of microelectrodes, continued innovation in materials and measurement strategies will be essential to fully realize their potential in advanced molten salt electrochemical systems.

## Appendix: List of Symbols

**Symbol Description**
*i*_ss_: steady-state current (A)
*i*_qss_: Quasi-steady-state current (A)
*i*_c_: capacitive charging current (A)
r: electrode radius (cm)
w: band width (cm)
A: electrode surface area (cm^2^)
C: bulk redox concentration (mol/cm^3^)
F: Faraday constant (96485.33C/mol)
D: diffusion coefficient (cm^2^/s)
n: number of electrons
t: time (s)
*C*_dl_: double-layer capacitance (F/cm^2^)
v: scan rate (V/s)

## References

[1] Serp J., Allibert M., Beneš O., Delpech S., Feynberg O., Ghetta V., Heuer D., Holcomb D., Ignatiev V., Kloosterman J. L. (2014). The Molten Salt Reactor (MSR) in Generation
IV: Overview and Perspectives. Prog. Nucl. Energy.

[2] Ding W., Bonk A., Bauer T. (2018). Corrosion
Behavior of Metallic Alloys
in Molten Chloride Salts for Thermal Energy Storage in Concentrated
Solar Power Plants: A Review. Front. Chem. Sci.
Eng..

[3] Salanne M., Simon C., Turq P., Madden P. A. (2008). Calculation of Activities
of Ions in Molten Salts with Potential Application to the Pyroprocessing
of Nuclear Waste. J. Phys. Chem. B.

[4] Kuravi S., Trahan J., Goswami D. Y., Rahman M. M., Stefanakos E. K. (2013). Thermal
Energy Storage Technologies and Systems for Concentrating Solar Power
Plants. Prog. Energy Combust. Sci..

[5] Vignarooban K., Xu X., Arvay A., Hsu K., Kannan A. M. (2015). Heat Transfer Fluids
for Concentrating Solar Power Systems – A Review. Appl. Energy.

[6] Guo S., Zhang J., Wu W., Zhou W. (2018). Corrosion in the Molten
Fluoride and Chloride Salts and Materials Development for Nuclear
Applications. Prog. Mater. Sci..

[7] Gill S. K., Sure J., Wang Y., Layne B., He L., Mahurin S., Wishart J. F., Sasaki K. (2021). Investigating Corrosion
Behavior of Ni and Ni-20Cr in Molten ZnCl2. Corros. Sci..

[8] Muránsky O., Karatchevtseva I., Danon A. E., Holmes R., Huai P., Zhang Z. (2020). Impact of
Dislocations and Dislocation Substructures on Molten Salt
Corrosion of Alloys under Plasticity-Imparting Conditions. Corros. Sci..

[9] Bawane K., Liu X., Gakhar R., Woods M., Ge M., Xiao X., Lee W.-K., Halstenberg P., Dai S., Mahurin S., Pimblott S. M., Wishart J. F., Chen-Wiegart Y. K., He L. (2022). Visualizing Time-Dependent
Microstructural and Chemical Evolution
during Molten Salt Corrosion of Ni-20Cr Model Alloy Using Correlative *Quasi in Situ* TEM and *in Situ* Synchrotron
X-Ray Nano-Tomography. Corros. Sci..

[10] Zhou W., Yang Y., Zheng G., Woller K. B., Stahle P. W., Minor A. M., Short M. P. (2020). Proton Irradiation-Decelerated
Intergranular
Corrosion of Ni-Cr Alloys in Molten Salt. Nat.
Commun..

[11] Suter T., Böhni H. (1997). A New Microelectrochemical
Method to Study Pit Initiation
on Stainless Steels. Electrochim. Acta.

[12] Compton, R. G. ; Banks, C. E. Understanding Voltammetry; World Scientific, 2018.

[13] Delpech S., Cabet C., Slim C., Picard G. S. (2010). Molten Fluorides
for Nuclear Applications. Mater. Today.

[14] Bard, A. J. ; Faulkner, L. R. ; White, H. S. Electrochemical Methods: Fundamentals and Applications; John Wiley & Sons, 2022.

[15] Corrigan D. K., Elliott J. P., Blair E. O., Reeves S. J., Schmüser I., Walton A. J., Mount A. R. (2016). Advances in Electroanalysis, Sensing
and Monitoring in Molten Salts. Faraday Discuss..

[16] Yang W., Lee N., Jung C., Park T.-H., Choi S., Bae S.-E. (2023). Microelectrode
Voltammetric Analysis of Samarium Ions in LiCl–KCl Eutectic
Molten Salt. Electrochem. Commun..

[17] Relf A., Corrigan D., Brady C. L., Terry J. G., Walton A. J., Mount A. R. (2013). Robust Microelectrodes in Molten
Salt Analysis. ECS Trans..

[18] Carlin R. T., Osteryoung R. A. (1989). Deposition Studies of Lithium and Bismuth at Tungsten
Microelectrodes in LiCl: KCl Eutectic. J. Electrochem.
Soc..

[19] Wang Y., Goh B., Sridharan K., Couet A. (2022). In Situ Corrosion Monitoring of the
T91 Alloy in a Molten Chloride Salt Using a Miniaturized Electrochemical
Probe for High-Throughput Applications. Anal.
Chem..

[20] Wang Y., Goh B., Moorehead M., Hattrick-Simpers J., Couet A. (2022). High-Throughput Electrochemistry
to Study Materials Degradation in Extreme Environments. Anal. Chem..

[21] Kim C., Couet A. (2025). In Situ Monitoring of Molten Chloride Salt Chemistry
and Corrosion
Using a Microelectrode. J. Am. Chem. Soc..

[22] Corrigan D. K., Blair E. O., Terry J. G., Walton A. J., Mount A. R. (2014). Enhanced
Electroanalysis in Lithium Potassium Eutectic (LKE) Using Microfabricated
Square Microelectrodes. Anal. Chem..

[23] Consiglio A. N., Carotti F., Liu E., Williams H., Scarlat R. O. (2022). Design
and Operation of a Molten Salt Electrochemical Cell. MethodsX.

[24] Williams T., Shum R., Rappleye D. (2021). Review—Concentration
Measurements
In Molten Chloride Salts Using Electrochemical Methods. J. Electrochem. Soc..

[25] Mejia C., Christensen N., Ceron R. R., Rappleye D. (2025). Development of a Stable
and Buffered Reference Electrode for Binary Molten Chlorides Salts. Electrochim. Acta.

[26] Grahame D. C. (1947). The Electrical
Double Layer and the Theory of Electrocapillarity. Chem. Rev..

[27] Zhang H., Choi S., Zhang C., Faulkner E., Alnajjar N., Okabe P., Horvath D. C., Simpson M. F. (2019). Square Wave Voltammetry
for Real Time Analysis of Minor Metal Ion Concentrations in Molten
Salt Reactor Fuel. J. Nucl. Mater..

[28] Ngamchuea K., Eloul S., Tschulik K., Compton R. G. (2014). Planar
Diffusion
to Macro Disc Electrodes—What Electrode Size Is Required for
the Cottrell and Randles-Sevcik Equations to Apply Quantitatively?. J. Solid State Electrochem..

[29] Forster R. J. (1994). Microelectrodes
New Dimensions in Electrochemistry. Chem. Soc.
Rev..

[30] Pletcher, D. Why Microelectrodes? In Microelectrodes: Theory and Applications; Montenegro, M. I. , Queirós, M. A. , Daschbach, J. L. , Eds.; Springer Netherlands: Dordrecht, 1991; pp 3–16.

[31] Puthongkham P., Venton B. J. (2020). Recent Advances
in Fast-Scan Cyclic Voltammetry. Analyst.

[32] Stojek Z., Osteryoung J. (1989). Experimental
Determination of the Coefficient in the
Steady State Current Equation for Spherical Segment Microelectrodes. Anal. Chem..

[33] Daniele, S. ; Denuault, G. From Microelectrodes to Scanning Electrochemical Microscopy. In Developments in Electrochemistry; John Wiley & Sons, Ltd, 2014; pp 223–244.

[34] Montenegro, I. ; Queirós, M. A. ; Daschbach, J. L. Microelectrodes: Theory and Applications; Springer Science & Business Media, 2012; Vol. 197.

[35] Billon G., van den Berg C. M. G. (2004). Gold
and Silver Micro-Wire Electrodes for Trace Analysis
of Metals. Electroanalysis.

[36] Guo Y., Chen D., Kim H., Shi F. (2025). Effect of Soluble Corrosion
Products on Electrical Double Layer in LiCl-KCl Molten Salts. J. Electrochem. Soc..

[37] Heinze J. (1993). Ultramicroelectrodes
in Electrochemistry. Angew. Chem., Int. Ed.
Engl..

[38] Verchère L., Aubert I., Devos O. (2019). Influence of the Crystallographic
Orientation on the Electrochemical Reactivity Measured by Scanning
Electrochemical Microscopy on Nickel-Based Alloy 600. Electrochim. Acta.

[39] Gateman S. M., Gharbi O., Turmine M., Vivier V. (2021). Measuring Changes in
Wettability and Surface Area during Micro Droplet Corrosion Measurements. Electrochim. Acta.

[40] Lucas, M. Microscopie Electrochimique En Milieu Sel Fondu: Experience, Modelisation et Application à l’étude de La Corrosion. PhD Thesis, Pierre and Marie Curie University (Paris 6), 2013.

[41] Okamoto H. (2009). Cr-Pt (Chromium-Platinum). J. Phase Equilib. Diffus..

[42] Popov A. A., Varygin A. D., Plyusnin P. E., Sharafutdinov M. R., Korenev S. V., Serkova A. N., Shubin Y. V. (2022). X-Ray Diffraction
Reinvestigation of the Ni-Pt Phase Diagram. J. Alloys Compd..

[43] Okamoto H. (2004). Fe-Pt (Iron-Platinum). J. Phs Eqil and Diff.

[44] Su X., Yin F., Huang M., Li Z., Chen C. (2001). Thermodynamic Assessment
of the Pt–Sn System. J. Alloys Compd..

[45] Lbibb R., Castanet R., Rais A. (2000). Thermodynamic
Investigation of Pt–Pb
Binary Alloys. J. Alloys Compd..

[46] McAlister A. J., Kahan D. J. (1986). The Al–Pt
(Aluminum-Platinum) System. Bull. Alloy Phase
Diagr..

[47] Hansen M., Anderko K., Salzberg H. W. (1958). Constitution
of Binary Alloys. J. Electrochem. Soc..

[48] Ciulik J., Notis M. R. (1993). The Au—Sn Phase Diagram. J. Alloys Compd..

[49] Lugovskoy A., Zinigrad M., Aurbach D., Unger Z. (2009). Electrodeposition of
Iron­(II) on Platinum in Chloride Melts at 700–750 °C. Electrochim. Acta.

[50] Duruz J. J., Stehle G., Landolt D. (1981). On the Role
of the Electrode Material
during Cathodic Deposition of Na and Al from Molten Fluorides. Electrochim. Acta.

[51] Issaeva L., Yang J., Haarberg G. M., Thonstad J., Aalberg N. (1997). Electrochemical
Behaviour of Tin Species Dissolved in Cryolite-Alumina Melts. Electrochim. Acta.

[52] Ye Z., Zhu Z., Zhang Q., Liu X., Zhang J., Cao F. (2018). In Situ SECM
Mapping of Pitting Corrosion in Stainless Steel Using Submicron Pt
Ultramicroelectrode and Quantitative Spatial Resolution Analysis. Corros. Sci..

[53] Etienne M., Dossot M., Grausem J., Herzog G. (2014). Combined Raman Microspectrometer
and Shearforce Regulated SECM for Corrosion and Self-Healing Analysis. Anal. Chem..

[54] Zhou Y., Sun L., Watanabe S., Ando T. (2022). Recent Advances in the Glass Pipet:
From Fundament to Applications. Anal. Chem..

[55] Cassayre L., Serp J., Soucek P., Malmbeck R., Rebizant J., Glatz J.-P. (2007). Electrochemistry
of Thorium in LiCl–KCl Eutectic
Melts. Electrochim. Acta.

[56] Yoon S., Kang D., Sohn S., Park J., Lee M., Choi S. (2020). Reference Electrode
at Molten Salt: A Comparative Analysis of Electroceramic
Membranes. J. Nucl. Fuel Cycle Waste Technol..

[57] Stern K. H. (1966). Glass-Molten
Salt Interactions. Chem. Rev..

[58] Dai S., Rodriguez M. A., Griego J. J. M. (2016). Sealing Glass-Ceramics with Near
Linear Thermal Strain, Part I: Process Development and Phase Identification. J. Am. Ceram. Soc..

[59] Dai S., Elisberg B., Calderone J., Lyon N. (2017). Sealing Glass-ceramics
with Near-linear Thermal Strain, Part III: Stress Modeling of Strain
and Strain Rate Matched Glass-ceramic to Metal Seals. J. Am. Ceram. Soc..

[60] Sehgal J., Ito S. (1999). Brittleness of Glass. J. Non-Cryst. Solids.

[61] Katasho Y., Yang X., Yasuda K., Nohira T. (2016). Electrochemical Reduction
Behavior of Borosilicate Glass in Molten CaCl2. J. Electrochem. Soc..

[62] Katasho Y., Yasuda K., Nohira T. (2017). Behaviors
of Si, B, Al, and Na during
Electrochemical Reduction of Borosilicate Glass in Molten CaCl2. J. Electrochem. Soc..

[63] Falconer C., Zhang H., Sridharan K., Couet A. (2023). Novel Pyrolytic Boron
Nitride Coating to Reduce Graphite Interactions in Molten Fluoride
Salt. Corros. Sci..

[64] Gunnell E., Blackwood B. L., Wilson C., Harb J. N., Memmott M. J. (2025). Investigation
of a U­(IV)/U­(III) Thermodynamic Reference Electrode for High-Temperature
Molten Fluoride Salts. J. Electrochem. Soc..

[65] Chan H. L., Singh H., Romanovski V., Romanovskaia E., Han J., Scully J. R. (2024). Uncovering Accurate
Values of the Polarization Resistance
in Molten Fluorides Using Electrochemical Impedance Spectroscopy. J. Electroanal. Chem..

[66] Huang V. M., Wu S.-L., Orazem M. E., Pébère N., Tribollet B., Vivier V. (2011). Local Electrochemical
Impedance Spectroscopy:
A Review and Some Recent Developments. Electrochim.
Acta.

[67] Polcari D., Dauphin-Ducharme P., Mauzeroll J. (2016). Scanning Electrochemical Microscopy:
A Comprehensive Review of Experimental Parameters from 1989 to 2015. Chem. Rev..

[68] Fan, F.-R. F. ; Liu, B. ; Mauzeroll, J. Scanning Electrochemical Microscopy. In Handbook of Electrochemistry; Elsevier, 2007; pp 471–540.

[69] Jorcin J.-B., Orazem M. E., Pébère N., Tribollet B. (2006). CPE Analysis
by Local Electrochemical Impedance Spectroscopy. Electrochim. Acta.

[70] Stull D. R. (1947). Vapor Pressure
of Pure Substances. Organic and Inorganic Compounds. Ind. Eng. Chem..

[71] Geng J., Luo Y., Fu H., Dou Q., He H., Ye G., Li Q. (2022). Temperature and Pressure
Effect on Evaporation Behavior of Chloride
Salts Using Low Pressure Distillation. Prog.
Nucl. Energy.

[72] Rocio Rodriguez Laguna, M. D. ; Karlsson, T. Y. Suggestions on the Vapor Pressure Determination of Molten Salts; Idaho National Lab.(INL), Idaho Falls, ID (United States), 2021. https://www.osti.gov/biblio/1826379 (accessed 2025–06–13).

[73] Jadhav N., Gelling V. J. (2019). Review—The Use of Localized Electrochemical
Techniques for Corrosion Studies. J. Electrochem.
Soc..

[74] Lillard R. S., Moran P. J., Isaacs H. S. (1992). A Novel
Method for Generating Quantitative
Local Electrochemical Impedance Spectroscopy. J. Electrochem. Soc..

[75] Zou F., Thierry D. (1997). Localized Electrochemical
Impedance Spectroscopy for
Studying the Degradation of Organic Coatings. Electrochim. Acta.

[76] Gharbi O., Ngo K., Turmine M., Vivier V. (2020). Local Electrochemical
Impedance Spectroscopy:
A Window into Heterogeneous Interfaces. Curr.
Opin. Electrochem..

[77] Weinstein M., Falconer C., Doniger W., Bailly-Salins L., David R., Sridharan K., Couet A. (2021). Environmental Degradation
of Electroplated Nickel and Copper Coated SS316H in Molten FLiNaK
Salt. Corros. Sci..

[78] Zhou H., Li Y., Morel A., Mauzeroll J. (2025). Chronopotentiometric Approach in
Scanning Electrochemical Cell Microscopy: Minimizing Surface Change
Upon Landing. ACS Appl. Mater. Interfaces.

[79] Tang X., Ma C. R., Orazem M. E., You C., Li Y. (2020). Local Electrochemical
Characteristics of Pure Iron under a Saline Droplet II: Local Corrosion
Kinetics. Electrochim. Acta.

[80] Li Y., Morel A., Gallant D., Mauzeroll J. (2020). Oil-Immersed
Scanning Micropipette Contact Method Enabling Long-Term Corrosion
Mapping. Anal. Chem..

[81] Goh B., Wang Y., Nelaturu P., Zhang H., Moorehead M., Duong T., Priya P., Thoma D., Chaudhuri S., Hattrick-Simpers J., Sridharan K., Couet A. (2024). Nobility vs. Mobility:
Insights into Molten Salt Corrosion Mechanisms of High-Entropy Alloys
via High-Throughput Experiments and Machine Learning. Matter.

